# Recent Progress in Ferrocene-Modified Thin Films and Nanoparticles for Biosensors

**DOI:** 10.3390/ma6125742

**Published:** 2013-12-06

**Authors:** Shigehiro Takahashi, Jun-ichi Anzai

**Affiliations:** Graduate School of Pharmaceutical Sciences, Tohoku University, Aramaki, Aoba-ku, Sendai 980-8578, Japan; E-Mail: t-shigehiro@m.tohoku.ac.jp

**Keywords:** ferrocene, biosensor, electron-transfer mediator, thin film, electro-polymerization, layer-by-layer deposition, Au nanoparticle

## Abstract

This article reviews recent progress in the development of ferrocene (Fc)-modified thin films and nanoparticles in relation to their biosensor applications. Redox-active materials in enzyme biosensors commonly use Fc derivatives, which mediate electron transfer between the electrode and enzyme active site. Either voltammetric or amperometric signals originating from redox reactions of Fc are detected or modulated by the binding of analytes on the electrode. Fc-modified thin films have been prepared by a variety of protocols, including *in* *situ* polymerization, layer-by-layer (LbL) deposition, host-guest complexation and molecular recognitions. *In* *situ* polymerization provides a facile way to form Fc thin films, because the Fc polymers are directly deposited onto the electrode surface. LbL deposition, which can modulate the film thickness and Fc content, is suitable for preparing well-organized thin films. Other techniques, such as host-guest complexation and protein-based molecular recognition, are useful for preparing Fc thin films. Fc-modified Au nanoparticles have been widely used as redox-active materials to fabricate electrochemical biosensors. Fc derivatives are often attached to Au nanoparticles through a thiol-Au linkage. Nanoparticles consisting of inorganic porous materials, such as zeolites and iron oxide, and nanoparticle-based composite materials have also been used to prepare Fc-modified nanoparticles. To construct biosensors, Fc-modified nanoparticles are immobilized on the electrode surface together with enzymes.

## 1. Introduction

**Figure 1:**
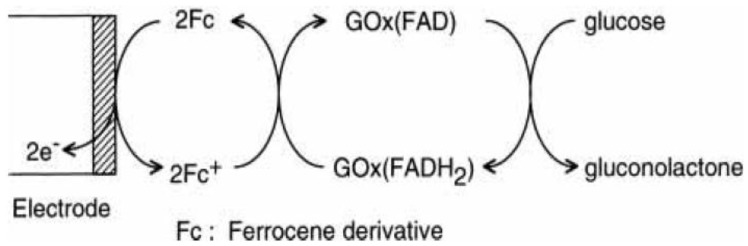
Ferrocene (Fc)-mediated electron transfer in amperometric glucose sensors. Reprinted with permission from Anzai *et al*. [8]. Copyright 2001 Pharmaceutical Society of Japan.

Redox-active materials, including small ions, molecules and polymers, are common electron-transfer mediators and electrochemical tags in biosensors. A wide range of materials are available for electron transfer, including iron and osmium complexes, hydroquinones, anthraquinones and organic dyes, such as methylene blue [1,2,3,4,5,6]. The corresponding polymers of such materials are also useful for mediator immobilization on an electrode surface. Among these materials, ferrocene (Fc) and Fc polymers are most commonly used in biosensor fabrication because of their high stability in redox reactions and facile derivative syntheses. Fc is a stable metal complex consisting of an iron(II) atom sandwiched between two cyclopentadienyl ligands. A typical example of Fc-mediated biosensors is an amperometric glucose sensor. Amperometric glucose sensors are constructed by immobilizing glucose oxidase (GOx) on the surface of metal or carbon electrodes, where the redox reaction of Fc is coupled with GOx -catalyzed glucose oxidation to mediate electron transfer between the active center of GOx and the electrode (Figure 1) [7,8]. Fc derivatives are usually confined on the electrode surface to construct reagentless biosensors, although dissolved Fc derivatives can also mediate electron transfer.

**Figure 2:**
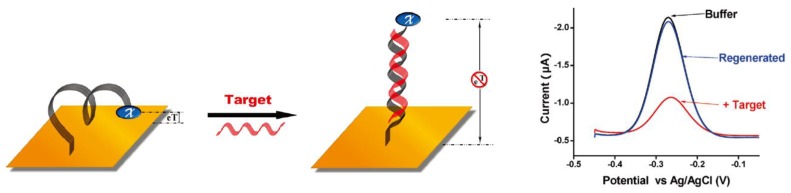
Fc-tagged DNA biosensor detecting hybridization. Reprinted with permission from White *et al*. [9]. Copyright 2009 American Chemical Society.

In other examples, Fc derivatives serve as redox-active tags for electrochemical determination of analytes. Typically, DNA-based sensors are constructed by modifying the electrode surface with single-stranded DNA chains with an Fc tag [9]. Redox reaction of the Fc tag depends on DNA chain hybridization, thus yielding a hybridization-dependent redox signal (Figure 2). An Fc-tagged aptamer has also been attached to the electrode surface [10]. In the examples described above, the design and fabrication of the Fc surface layer on the electrode is crucial for modulating the sensor response characteristics. Consequently, many protocols have been developed for constructing Fc-modified surface layers on biosensor electrodes. In this context, much research has recently focused on developing highly ordered thin films and nanoparticles. In this review, we focus on recent progress in the development of Fc-modified thin films and nanoparticles for biosensor applications.

## 2. Fc-Containing Thin Films

Fc-modified polymers have been widely used to form thin films on electrode surfaces for fabricating mediator-type biosensors. Three different strategies are often employed in the preparation of Fc thin films: *in* *situ* polymerization, layer-by-layer (LbL) deposition and selective adsorption.

### 2.1. In Situ Polymerized Fc Films

*In* *situ* polymerization is a facile way to form thin films on electrode surfaces, because it circumvents difficulties in chemical synthesis and polymer purification. *In* *situ* synthesized polymers are usually deposited on the electrode surface during polymerization. Plasma polymerization [11,12], photopolymerization [13,14], chemical polymerization [15] and electropolymerization [16,17,18,19,20,21,22,23,24,25,26,27] have all been used in this context. Muguruma and Uehara reported excellent plasma-polymerized film properties, including good substrate adhesion, a flat pinhole-free surface and high mechanical and chemical stability [11,12]. They used plasma polymerization to prepare thin films of dimethylaminomethylferrocene on the surface of a GOx -modified electrode. A highly effective electron-mediation system was constructed in the glucose sensors. An advantage of plasma polymerization is that the entire process is carried out in the vapor phase. Therefore, plasma-polymerized films are suitable for high-throughput production of microscale bioelectronic devices. Heng and coworkers reported a single-step glucose sensor fabrication protocol based on photochemically polymerized films consisting of Fc derivatives and GOx [13,14]. A mixed solution of 2-hydroxyethyl methacrylate, Fc derivative, initiator and GOx was deposited on the surface of a screen-printed carbon electrode and irradiated with UV light. Unsubstituted Fc is physically trapped in the film, whereas vinylferrocene is covalently immobilized. The main advantage of this procedure is that biosensors can be constructed in a single step without any pre- or post-treatment with enzymes. A conventional polymerization procedure is also useful for modifying an electrode surface with Fc thin films [15].

Electropolymerized conductive polymers have attracted considerable attention in the development of electrochemical sensors and reactors [16,17,18,19,20,21,22,23]. Conductive polymer films can be deposited on electrode surfaces by using heterocyclic compounds, such as pyrroles [16,17,18,19], thiophenes [20,23] and anilines [23] as monomers. An advantage of electrochemical polymerization procedures is that regulating the applied electrode potential and/or electric current readily controls film formation. Film thickness can be controlled by changing the electrolysis time. Moreover, functional biomolecules, such as proteins and nucleic acids, can be entrapped in electropolymerized films if the biomolecules are dissolved in the monomer solution during electropolymerization [16,21]. Considerable flexibility in monomer types enables the modulation of the films’ electrochemical properties. A variety of monomeric structures, including Fc derivatives, have been synthesized for improving the conductivity and electron-transfer efficiency of the films. Fc-substituted thiophene and terthiophene have been used as monomers for preparing conducting polymers on a Pt electrode [24]. The polymer-modified Pt electrode facilitates electron transfer to cytochrome C, suggesting the potential use of the electrode in biosensors. Fang and coworkers used Fc-substituted cationic polythiophene, which was prepared via chemical polymerization, as a redox marker for detecting DNA hybridization on the surface of peptide nucleic acid (PNA)-modified electrodes [25]. Fc-substituted polythiophene is electrostatically bound to only the PNA-DNA hybridization product, yielding an electrochemical signal in the presence of target DNA chains, whereas no signal is observed for four-base mismatch DNA. Leclerc and coworkers used aggregates consisting of Fc-substituted polythiophene as a redox marker for ultrasensitive determination of target DNA chains (the detection limit of the target DNA was 4 × 10^−16^ M) [26]. The utility of polymeric redox markers in sensitive DNA detection has been reported [27]. Şenel recently proposed reagentless glucose biosensors based on Fc-substituted polypyrrole films [28]. The author modified the films with GOx and electrochemically deposited them on the electrode surface. The Fc moieties in the films served as electron-transfer mediators between GOx and the electrode.

### 2.2. LbL-Deposited Fc Films

The LbL deposition technique, commonly used for constructing thin films and nanoscale assemblies [29,30,31,32,33,34,35], is a facile way to form polymer thin films by alternating, repeated adsorption of cationic and anionic polymers onto the substrate from aqueous solution. Such thin films are common in thin-film devices because of the wide array of useful polymer types, including biopolymers, such as proteins [36,37], polysaccharides [38,39] and polypeptides [40]. LbL film-modified devices are used in biosensors [41], ion-selective membranes [42], stimuli-sensitive systems [43] and drug delivery [44]. Early research demonstrated that Fc-containing LbL films are useful for fabricating mediator-type biosensors and electrocatalysts [45,46,47,48,49,50,51]. For example, Calvo and coworkers prepared glucose biosensors by using an Au electrode coated with LbL films consisting of Fc-bearing poly(allylamine) (Fc-PAH) and GOx [45]. The Fc moieties in the LbL film effectively mediated electron transfer between GOx and the electrode, depending on the film thickness and configuration. Sun and coworkers improved Fc-PAH/GOx LbL film-modified glucose sensors by using periodate-oxidized GOx to covalently connect the LbL layers through Schiff-base formation [46]. Copolymers consisting of vinylferrocene and (methacryloyloxy)ethyl trimethylammonium units have also been used to construct LbL films [47]. Our laboratory has prepared Fc-containing LbL film-coated electrodes [48,49,50]. The redox properties of the Fc LbL film-coated electrodes are dependent on the polymer type, Fc content in the polymer and LbL film configuration. We used our Fc-PAH film-coated electrodes for solution-phase electrocatalytic determination of ascorbic acid. Fc-polymer films containing DNA may also be useful for sensing intercalating drugs [51]. An advantage of LbL films arises from facile regulation of the thickness and multilayer structure of the film, facilitating the film-structure design at the molecular level and, thus, performance optimization.

**Figure 3:**
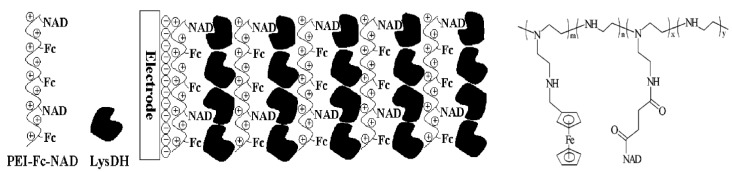
A reagentless L-lysine sensor prepared using Fc-(nicotinamide adenine dinucleotide)-bearing poly(ethylenimine). Reprinted with permission from Zhang *et al*. [55]. Copyright 2008 Wiley-VCH. NAD, nicotinamide adenine dinucleotide; PEI, poly(ethylenimine).

Zheng and Suye’s group developed enzyme and DNA biosensors using Fc polymers [52,53,54,55]. They incorporated L-proline dehydrogenase and Fc-PAH into the LbL film through electrostatic affinity to construct L-proline sensors [54]. The catalytic current of the sensor in response to L-proline increased as the number of Fc-PAH layers in the LbL film was increased. For preparing L-lysine sensors, Zheng and Suye’s group synthesized a poly(ethylenimine) (PEI) derivative bearing coenzyme (nicotinamide adenine dinucleotide, NAD) and Fc moieties. They alternately deposited the Fc-NAD-bearing PEI and L-lysine dehydrogenase on an Au electrode to prepare reagentless L-lysine sensors, in which all the components (enzyme, coenzyme and electron-transfer mediator) were confined in the LbL film (Figure 3) [55]. The L-lysine sensor exhibits an electrochemical response to L-lysine in the concentration range of 1–120 mM. Fc-PAH and Fc-PEI are often used as the cationic component in preparing electrostatic LbL films. In this way, negatively charged enzymes are successfully combined with the Fc polymers to form LbL films for biosensors. Similarly, Fc-PEI has been used to fabricate Au nanoparticle (AuNP)/Fc-PEI LbL films [56,57]. UV-Vis absorption spectroscopy and atomic force microscopy clearly show a layered structure in the AuNP/Fc-PEI films [56]. The AuNP/Fc-PEI film-coated electrodes exhibit electrocatalytic reactions (ascorbic acid oxidation and oxygen reduction). Dong and Wang’s group used AuNP/Fc-PEI film-coated electrodes to construct aptamer sensors for cocaine [57]. They modified AuNPs on the surface of a (AuNP/Fc-PEI)_2_ film-coated electrode via a cocaine-sensitive aptamer fragment. The redox current originating from the Fc moieties in the LbL film decreases in the presence of aptamer and cocaine (*i.e*., in accordance with cocaine concentration) in the range of 0.1–88.8 µM. They attribute the reduced redox current to the reduced apparent diffusion coefficient of the Fc moiety in the film. This strategy has wide applicability in fabricating reagentless affinity sensors, although the response mechanism of the sensors must be further studied. This system is applicable to thrombin and lysozyme determination [57,58].

**Figure 4:**
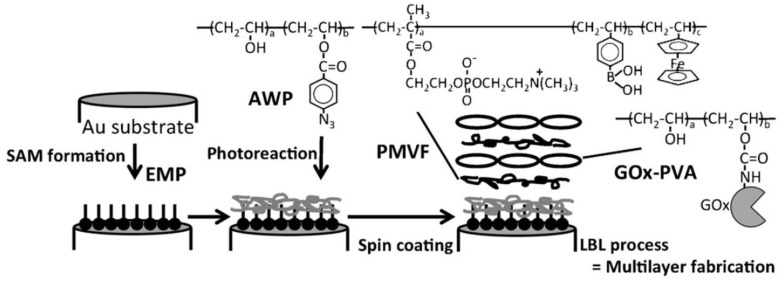
A glucose sensor prepared using phosphorylcholine polymers containing an Fc unit. Reprinted with permission from Ishihara *et al*. [59]. Copyright 2012 Elsevier. LbL, layer-by-layer.

Different types of Fc polymers have been used as components of redox-active LbL films. Ishihara and coworkers used phosphorylcholine polymers containing an Fc unit to prepare a multilayered hydrogel on an electrode surface to fabricate glucose sensors (Figure 4) [59]. Vancso and coworkers prepared water-soluble ferrocenylsilane polymers that have positive or negative charges [60,61]. These Fc polymers contain Fc moieties in their main chains, as opposed to pendant Fc moieties in Fc-PAH and Fc-PEI. They incorporated ferrocenylsilane polymers into LbL films and microcapsules, the thickness, swelling and permeability of which are tunable through redox reactions of the Fc residues [62,63,64]. Their Fc polymer-modified electrode is useful for electrocatalytic determination of ascorbic acid [65].

### 2.3. Host-Guest Complexation and Molecular Recognition

**Figure 5:**
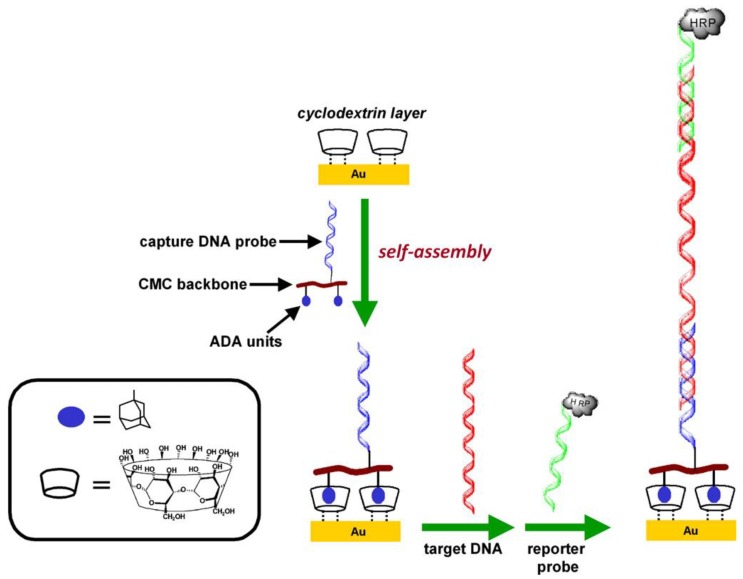
Genosensor fabrication based on host-guest complexation on an electrode surface. Reprinted with permission from O’Sullivan *et al*. [68]. Copyright 2011 Elsevier.

Fc and Fc polymers can be immobilized on the surface of electrodes through host-guest complexation. Cyclodextrins (CDs) are cyclic oligosaccharides that form inclusion complexes with hydrophobic molecules. CDs are commonly used for forming thin films and chemically modifying electrode surfaces [66,67,68,69]. Marken and coworkers deposited carboxymethyl-γ-CD and mesoporous TiO_2_ nanoparticles onto the surface of an indium tin oxide (ITO) electrode to form thin layers [70]. The 1,1ʹ-ferrocenedimethanol (DMFc) complex with carboxymethyl-γ-CD exhibits characteristic redox signals on the ITO surface. However, the DMFc is released from the electrode surface upon oxidation because of weak binding of oxidized DMFc to the CD. On the other hand, the Fc polymer is firmly immobilized on the surface of the CD monolayer-modified electrode. O’Sullivan and coworkers fabricated genosensors based on host-guest complexation on a CD monolayer-modified electrode (Figure 5) [68]. The multipoint binding between the DNA-modified carboxymethylcellulose (CMC) and CD enables stable anchoring of the CMC derivative on the surface, despite the fact that the 1:1 binding to CD is relatively weak. In another report, the same group synthesized CMC derivatives, dually modified with Fc and DNA chains, to fabricate DNA sensors [71]. They immobilized the Fc-DNA-modified CMC on the surface of a CD monolayer-modified electrode through host-guest complexation. Thus, the Fc moiety served as redox-active sites, as well as anchoring sites on the electrode surface. In this context, Fc polymers were incorporated into thin films and microcapsules through CD complexation [72,73]. These Fc-containing materials are expected to be useful for fabricating electrochemical biosensors.

Binding proteins, such as avidin and lectin, are also useful tools for immobilizing functional molecules on solid surfaces [33,74,75,76,77,78]. Tiefenauer and coworkers prepared Fc-labeled avidin for assembling protein architectures, to which they coupled biotinylated enzymes to fabricate biosensors [79,80]. They used avidin as a linker for immobilizing biotinylated alkaline phosphatase on the surface of biotin-terminated Fc monolayer films [81]. They studied the voltammetric properties of Fc-sugar and Fc-glycogen conjugates in the presence and absence of lectin [82,83,84,85]. The Fc conjugates will be used to construct sugar sensors. Fc-labeled aptamers are also useful for fabricating electrochemical biosensors [86,87].

### 2.4. Miscellaneous

Interesting procedures for immobilizing Fc on electrode surfaces, based on click chemistry [88,89] and boronate ester formation [90], have recently been reported. A Au electrode was modified with an azide-terminated self-assembled monolayer, to which alkyne-substituted Fc was added via a click reaction in the presence of Cu^2+^ catalyst [88]. The corresponding Fc-modified electrode exhibited a strong response to ascorbic acid in the range of 5.0 × 10^−12^ to 1.0 × 10^−9^ M. Alternatively, azide-substituted Fc can be confined to an alkyne-modified surface [89]. Fc-boronic acid was immobilized on an electrode surface via boronate ester formation, in which a tyrosine-modified electrode was treated with tyrosinase in advance to generate catechol functionality on the surface [90]. Thus, the extent of Fc-boronic acid immobilization or the magnitude of the redox signal depends on the catalytic activity of tyrosinase, enabling tyrosinase determination. Other papers report the use of Fc-boronic acid as a redox marker in the voltammetric determination of sugars, phenols and glycated hemoglobin [91,92,93,94,95].

Fc-PEI is commonly used to prepare amperometric biosensors because of its desirable properties, such as high segmental mobility of the polymer chain, facile control of Fc substitution degree and high swellability in water [96]. Glatzhofer and Schmidtke’s group developed high-performance glucose and H_2_O_2_ sensors using Fc-modified linear PEI [97]. Their linear Fc-PEI-based sensors enable glucose detection in the micromolar range. They evaluated the effects of Fc methylation in the Fc-PEI complex [98]. Dimethyl- and tetramethyl-substituted Fc polymers were identified as promising redox components for biofuel cells and biosensors. Furthermore, Fc-modified polysiloxane has been used to construct glucose biosensors [99]. The redox potential of the Fc moiety in the polymer shifts in the negative region as a result of composite formation with chitosan. The composite is a useful scaffold for enzyme immobilization. Fc-tethered poly(amidoamine) dendrimers (Fc-D) have been employed for constructing a biosensor interface on electrodes [100]. Fc-D can be immobilized on the surface of a carboxyl-terminated monolayer-modified Au electrode through an amide linkage, on which periodate-oxidized GOx is assembled through Schiff-base formation. Electron-transfer mediation through the Fc-D layer depends on the linker length between the Fc residues and the dendrimer. Reviews on recent progress in biomedical and biosensor applications of dendrimers are available [101,102].

Fc thin films sometimes suffer from instability in the redox reaction. Cross-linked Fc-PEI films are unstable upon repeated cyclic voltammetry scanning in phosphate solutions at pH 7.0, whereas the film is stable at pH 5.0 [103]. The peak current in cyclic voltammetry decreases with increasing scan number. In contrast, the redox reaction is stable in a perchlorate solution at pH 7.0, although there is instability at pH 11. This is attributable to the instability of ferrocenium (oxidized Fc), which decomposes in the presence of nucleophiles. The instability of Fc-modified films has been studied in relation to the chemical structure of the polymer backbone. Bunte and Rühe synthesized two different types of poly(acrylic acid) derivatives bearing Fc side chains [104]. The Fc polymer is unstable at pH 7.2 if the polymer contains secondary amino groups in the side chains connecting Fc to the polymer backbone, whereas there is a stable redox reaction for an Fc polymer with poly(ethylene oxide) side chains. These results suggest that the instability of Fc thin films arises from nucleophilic anion uptake in the film through amino-group binding.

## 3. Fc-Modified Nanoparticles

Nanoparticles consisting of metals [105,106,107,108], metal oxides [107,109], metal complexes [110] and polymers [111] are generating much interest, because of their attractive optical and electronic properties, as well as their potential applications in a wide variety of fields, such as biosensor development. In the following sections, we discuss Fc-containing nanoparticles employed in biosensor assemblies, in which Fc derivatives are attached to the nanoparticles to improve the electron-transfer properties of Fc.

### 3.1. Fc-Modified Au Nanoparticles

**Figure 6:**
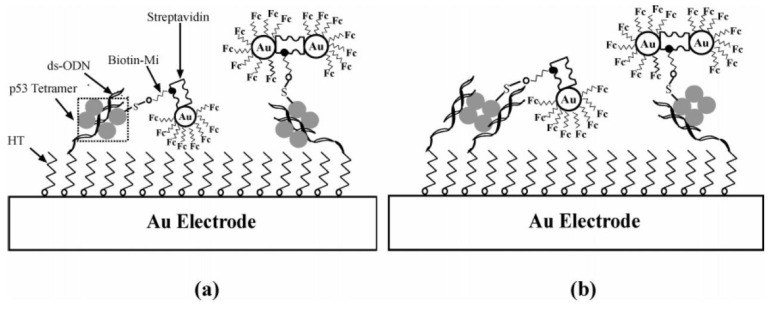
p53 protein sensors based on Fc-modified AuNP/streptavidin conjugates. (**a**) shows the most stable p53 binding mode on the sensor electrode; while (**b**) depicts two other possible binding configurations. Reprinted with permission from Zhou *et al*. [117]. Copyright 2011 American Chemical Society.

AuNPs are commonly used as the core material to immobilize Fc derivatives, owing to the ease of surface modification with thiol compounds. Self-assembled monolayer films based on the thiol-Au linkage are commonly used for modifying Au electrode surfaces in biosensor fabrication [3,112,113]. Zhou and coworkers prepared Fc-modified AuNP/streptavidin conjugates using 6-ferrocenylhexanethiol and used their conjugates for voltammetric detection of DNA hybridization and proteins [114,115,116,117]. They attached AuNP/streptavidin conjugates to the electrode surface through streptavidin-biotin linkages. The electrode response was strongly enhanced, because AuNP/streptavidin conjugates contain 127 ± 18 Fc residues per AuNP, and one or two AuNPs are attached to each streptavidin molecule. A tumor-suppressor protein, p53, can be detected at concentrations as low as 2.2 × 10^−12^ M (Figure 6) [117]. Liu and Xia’s group used Fc-modified AuNP/streptavidin conjugates for voltammetric determination of dopamine [118]. AuNPs co-modified with DNA and Fc have been prepared by using thiolated DNA and 6-ferrocenylhexanethiol; these AuNPs are used as a redox marker in voltammetric evaluation of site-specific DNA cleavage [119]. In another protocol, an Fc/6-thio-β-cyclodextrin inclusion complex was attached to AuNPs to construct amperometric glucose sensors [120]. Fc-modified Au nanoparticles were built into LbL architectures by alternating deposition of AuNPs and poly(diallyldimethylammonium chloride) [121]. Glassy carbon electrodes modified with the LbL film exhibit a catalytic response to ascorbic acid in the concentration range of 8 × 10^−6^ to 6 × 10^−3^ M. Escorcia and Dhirani fabricated an LbL film-coated electrode based on direct deposition of AuNPs and a dithio-substituted Fc derivative [122]. Voltammetric studies on the LbL film-coated electrodes suggest a disordered and porous structure of the LbL layer. Yuan and coworkers used AuNPs modified with Fc-labeled antibodies to prepare electrochemical immunosensors [123]. In all the research we have presented in this section, Fc derivatives were affixed to AuNPs via thiol-Au interactions.

### 3.2. Fc-Modified Inorganic Porous Materials

Inorganic porous materials, for example, CaCO_3_, zeolite, silica and iron oxide (Fe_3_O_4_) particles, have been used as supports for immobilizing proteins in biosensor assemblies [124,125,126,127]. Their porous nature enables many proteins to bind to the surface. Li and Yang reported a facile protocol for preparing Fc-modified CaCO_3_ particles [128], in which they dispersed CaCO_3_ particles, positively and negatively charged polymers and Fc in a methanol/water solution that they stirred at 60 °C until the solution evaporated. This simple procedure immobilizes the Fc and polymers on the surface of the CaCO_3_ particles. They further modified the CaCO_3_ particles with an antibody to fabricate immunosensors sensitive to interleukin-6. In another report, Fc was firmly encapsulated via sublimation in the cavities of a NaY zeolite through vapor diffusion [129]. The encapsulated Fc is electrochemically active and does not leak out of the cavity. The Fc-modified zeolite and GOx were immobilized on the electrode surface to fabricate glucose biosensors. Silica nanoparticles (approximately 15 nm in diameter) have been modified with Fc to fabricate glucose biosensors [130]. The surface of Fe_3_O_4_ particles is readily modified with Fc carboxylic acid via a dopamine bridge [131] and cyclodextrin complexation [132]. Dopamine is noteworthy as a stable modifier for Fe_3_O_4_ particles to further introduce functional molecules at the surface [133].

### 3.3. Fc-Modified Composite Nanoparticles

Composite nanoparticles are also useful as core materials for Fc complex assembly. Au-containing composites have been modified with Fc derivatives by taking advantages of thiol-Au binding. Luong and coworkers employed Au-carbon nanotube composites for immobilizing an Fc thiol derivative on an electrode surface [134,135]. The modified electrodes are useful for screening HIV-1 protease activity. A Au-TiO_2_ composite has been used to immobilize an Fc derivative for fabricating glucose biosensors [136].

**Figure 7:**
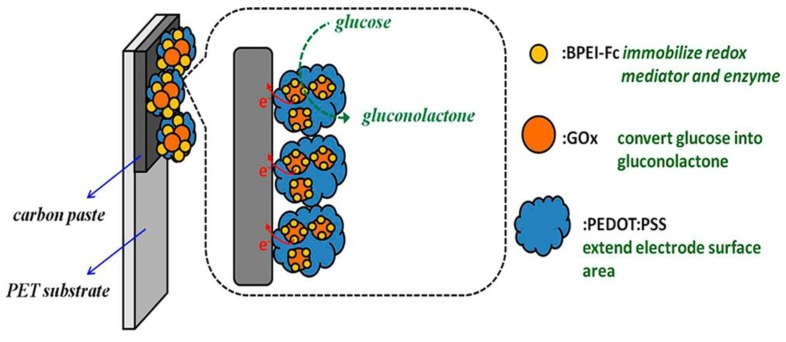
Redox polymer nanobeads composed of Fc-modified PEI for glucose biosensors. Reprinted with permission from Ho *et al*. [151]. Copyright 2010 American Chemical Society.

Carbon nanotubes have widely been used as a conducting material in biosensor assemblies [137,138,139,140,141]. Pyrene-substituted Fc has been affixed to carbon nanotubes through π-π stacking [142]. This strategy is valuable, because any type of pyrene-bearing compounds can be attached to carbon nanotubes through such stacking. A variety of protocols are available for preparing Fc-modified carbon nanotubes, which include bridging through amide [143] and Schiff-base bonding [144]. The surface of azide-bearing carbon nanotubes has been modified with alkyne-substituted Fc through click chemistry [145]. LbL deposition of negatively charged carbon nanotubes and either Fc-PAH or Fc-PEI yields biosensor platforms on electrode surfaces [146,147]. The surface of the LbL films was further modified with folic acid to recognize cancer cells specifically, enabling highly sensitive determination of human cervical carcinoma cells. There are potential uses for Fc-modified graphene and carbon nanoparticles in biosensor fabrication [148,149,150]. Fc-PEI nanobeads have been synthesized and mixed with GOx for drop-coating on a carbon electrode in order to develop amperometric glucose sensors (Figure 7) [151]. The Fc-PEI nanobeads were further modified with a conductive polymer, poly(3,4-ethylenedioxythiophene), to enhance the glucose sensor’s performance.

## 4. Conclusions

Fc derivatives can be successfully assembled into thin films and nanoparticles for the development of high-performance biosensors. Fc-modified thin films can be prepared on electrode surfaces by *in situ* polymerization, LbL deposition, host-guest complexation and molecular recognition. Nanoparticles consisting of metal oxides, metal complexes or polymers can be modified with Fc. Both Fc-modified thin films and nanoparticles exhibit excellent electron transfer and signal modulation in biosensors. The reasonable stability of Fc in redox reactions is advantageous in biosensor applications of Fc-modified thin films and nanoparticles, though, in some cases, improvement in the stability of Fc is further required. Consequently, Fc-modified thin films and nanoparticles are expected to find further applications in biosensors.
